# The Effect of Multivitamin/Mineral Supplements on Age-Related Cataracts: A Systematic Review and Meta-Analysis

**DOI:** 10.3390/nu6030931

**Published:** 2014-02-28

**Authors:** Li-Quan Zhao, Liang-Mao Li, Huang Zhu, The Epidemiological Evidence-Based Eye Disease Study Research Group

**Affiliations:** 1 Department of Ophthalmology, No. 181 Hospital of the PLA, No.1 Xinqiaoyuan Road, Guilin 541002, China; E-Mail: llm3891288@163.com; 2 Department of Ophthalmology, Xinhua Hospital Affiliated to Shanghai Jiao Tong University School of Medicine, No. 1665 Kongjiang Road, Shanghai 200092, China; E-Mail: zhuwjp@sh163.net

**Keywords:** dietary supplements, cataract, vitamins, minerals, meta-analysis

## Abstract

Antioxidant vitamins supplements have been suggested as a strategy to decrease the risk of age-related cataract development. However, the results from observational studies and interventional trials of associations between antioxidant vitamins A, C, and E and cataract development have been inconsistent. We aim to evaluate the effectiveness of multivitamin/mineral supplements for decreasing the risk of age-related cataracts by conducting a systematic review and meta-analysis. In September 2013, we searched multiple databases to identify relevant studies including both cohort studies and randomized controlled trials (RCTs). A random-effects model was used to calculate the pooled relative risks (RR) with a 95% confidence interval (CI). Twelve prospective cohort studies and two RCTs were included. Pooled results from the cohort studies indicated that multivitamin/mineral supplements have a significant beneficial effect in decreasing the risk of nuclear cataracts (RR: 0.73; 95% CI: 0.64–0.82), cortical cataracts (RR: 0.81; 95% CI: 0.68–0.94), and any cataracts (RR: 0.66; 95% CI: 0.39–0.93). In addition, there were no decreases in the risk of posterior capsular cataracts (RR: 0.96; 95% CI: 0.72–1.20) or cataract surgery (RR: 1.00; 95% CI: 0.92–1.08). The two RCTs demonstrated that multivitamin/mineral supplements could decrease the risk of nuclear cataracts. There is sufficient evidence to support the role of dietary multivitamin/mineral supplements for the decreasing the risk of age-related cataracts.

## 1. Introduction

Age-related cataracts are the main cause of the loss of useful vision among the elderly population, with an estimated 16 million people affected worldwide [1]. Oxidative stress is widely accepted to be a significant factor in the genesis of age-related cataracts [2,3]. Therefore, antioxidant vitamin supplements have been proposed as a strategy to decrease the risk of age-related cataracts [4,5]. *In vitro* laboratory studies and animal experiments have shown that antioxidants such as vitamin E, vitamin C, and β-carotene reduce oxidative stress, lipid peroxidation, and free radical damage, which are important intermediate steps in the pathogenesis of age-related cataracts [6,7].

Over the past two decades, many observational epidemiological studies have reported associations between diet and cataract development, particularly for the antioxidant vitamins A, C, E, lutein, and zeaxanthin [8,9,10,11,12,13,14,15,16,17,18,19,20,21,22,23,24,25,26,27]. These studies yielded inconsistent results. Both randomized controlled trials (RCTs) and a meta-analysis of these RCTs have found no evidence that supplementation with antioxidant vitamins (β-carotene, vitamin C, or vitamin E) prevents or slows the progression of age-related cataracts [28,29,30,31,32,33,34,35,36,37,38].

In contrast to taking one or several main antioxidant supplements, the use of multivitamin/mineral supplements is more popular among the general population, as confirmed by many population-based surveys [39,40,41,42]. Many of the observational studies reporting associations between the dietary intake of antioxidants from food and supplements and the risk of cataract development are population-based cohort studies, such as The Blue Mountains Eye Study [10] and The Beaver Dam Eye Study [8,9]. The findings of these studies regarding the association between multivitamin/mineral supplements and the risk of cataract development are possibly pertinent to this public health issue and have not yet been fully utilized in data synthesis.

We completed a systematic review and meta-analysis to provide pooled quantitative estimates of the likely magnitude of the effect of multivitamin/mineral supplements on clinical cataract outcomes. This report forms part of a large systematic review and meta-analysis of the existing literature on the association between antioxidants and the risk of cataract formation in humans.

## 2. Methods

### 2.1. Data Sources

We developed a search strategy to identify studies that investigated the association between multivitamin/mineral supplements and cataract risk. We searched the electronic databases PUBMED, EMBASE, and Cochrane Central Register of Controlled Trials for literature published until 1 September 2013. For maximum sensitivity, we used free text and Mesh terms including “diet OR intake OR supplement OR food OR nutrient OR antioxidant OR micronutrient OR vitamin OR trace elements OR trace minerals” AND “cataract OR age-related cataract OR lens opacity OR cataract surgery OR clinical cataract events”. For those titles and/or abstracts that met these criteria, the full article was retrieved. The search was conducted to identify all published studies of the relationship between multivitamin/mineral supplements and age-related cataracts. RCTs and prospective cohort studies were all included. The reference lists of the identified relevant studies and previous reviews were manually checked for further relevant studies. No language restrictions were applied.

### 2.2. Study Selection

We selected studies if they met the following inclusion criteria: (1) clinical trials in humans that assessed the preventive effect of multivitamin/mineral combination supplements; (2) studies that reported clinical outcomes that were closely associated with clinical cataract events, including any lens opacity, nuclear opacity, cortical opacity, posterior subcapsular opacity, and cataract surgery; (3) studies that were designed as RCTs or as prospective cohort studies; and (4) studies that reported original data about the risks of clinical cataract events, including odds ratios, risk ratios, relative risks, or hazard ratios and their 95% confidence intervals (CIs) with respect to some form of multivitamin/mineral supplement.

The exclusion criteria were the following: studies in which the outcomes were not clearly defined; and review letters, commentaries, and editorials not containing original data.

When multiple publications of the same study existed, we included only the publication containing the most detailed information regarding both the outcomes and antioxidant vitamin supplements. When the same study generated several different reports, we used the findings of the report that included all the participants or the report with the longest follow-up period.

### 2.3. Data Extraction and Study Quality

Data extraction and evaluation of the studies’ quality were completed independently by two reviewers (LQZ and HZ). The data were recorded onto a customized data extraction form described in the Cochrane Handbook for Systematic Reviews of Interventions, which included each study’s author, year of publication, location, patient characteristics, sample size, mean age or age range of subjects, sex of subjects, methods of measuring multivitamin/mineral supplements, and covariates used for adjustments in multivariate models.

The quality of cohort studies was assessed using the Newcastle-Ottawa Scale for Quality Assessment (NOS) [43]. The reviewers examined the following points of methodological quality: selection, comparability, and exposure or outcome. The quality of RCTs was assessed using the *Cochrane Handbook for Systematic Reviews of Interventions* [44]. The reviewers examined the following points of methodological quality: allocation concealment, method of allocation of treatment, masking of outcome assessment, and completeness of follow-up. The scores from these instruments were categorized as high, moderate, or low quality.

The two reviewers resolved any disagreement about study inclusion, quality assessment, selection of outcome parameters, and data extraction through discussion until full agreement was reached.

### 2.4. Data Analysis

For the observational studies, the outcome parameters investigated were the pooled relative risk (RR) with a 95% CI for clinical cataract events, comparing the longest duration of multivitamin/mineral supplementation with the shortest duration or with no multivitamin/mineral supplementation. Measures of association (odds ratios, relative risks, risk ratios, or hazard ratios) and their 95% CIs were abstracted or derived using data reported in the publications.

To pool RR estimates from individual studies, we used an inverse-variance weighted random-effects model. Wherever possible, risk estimates from the most fully adjusted models were used in the estimation of the pooled RR. We presented the more conservative pooled RR estimates generated by the inverse-variance weighted random effects models rather than those generated by the fixed effects models because of the different pre-existing conditions involved in the original trials. Clinical trials were analyzed according to the intention to treat principle.

We used the *I*^2^ index to estimate the heterogeneity existing among the studies. Values of 25%, 50%, and 75% represented low, moderate, and high degrees of heterogeneity, respectively. Pre-specified subgroup analyses were conducted to explore the potential sources of heterogeneity existing among the studies when there were sufficient studies to analyze study design (such as population-based studies), study quality, gender differences, and supplement duration.

Finally, we assessed publication bias using funnel plots and Begg’s tests. Funnel plots were produced and checked for asymmetries to ascertain whether a publication bias was present.

The systematic review was performed in accordance with the MOOSE guidelines. The PRISMA statement checklist was used [45]. Statistical analyses were performed using STATA version 12 (STATA Corp., College Station, Brazos, TX, USA); *P* values less than 0.05 were considered statistically significant.

## 3. Results

### 3.1. Overview of Included Studies

Of the initial 2704 papers identified by the literature search, 135 had potentially eligible abstracts and were retrieved for further scrutiny. Of these, twelve prospective cohort studies and two RCTs had available outcome data and met the selection criteria [8,9,10,11,12,13,14,15,16,17,18,19,46,47].

Among the observational studies, two were from The Beaver Dam Eye Study [8,9], one was from The Blue Mountains Eye Study [10], one was from The Swedish Mammography Cohort [11], one was from The Cohort of Swedish Men [12], one was from The Age-Related Eye Disease Study (AREDS) [13], one was from The Longitudinal Study of Cataract [14], one was from The Physicians’ Health Study [15], and four were from The Nurses’ Health Study [16,17,18,19]. The two RCTs were from The Clinical Trial of Nutritional Supplements and Age-Related Cataract Study [46] and The Linxian Cataract Study [47].

Among these ten independent prospective cohort studies and RCTs, six were from the United States, two from Sweden, one from Australia, and one from China. Two studies exclusively enrolled men, and two studies exclusively enrolled women. The number of subjects enrolled in the studies ranged from 478 to 50828. The age of the participants was older than 40 years. The follow-up was 4.8–15 years. The first article reporting data was published in 1992.

Further information about the individual studies is presented in Table 1 and Table 2.

**Table 1:** Prospective cohort studies evaluating multivitamin/mineral supplementation and its association with the low risk of age-related cataract.

Author, Year (Location)	Study Name	Follow-Up Time (Year)	Population (Number, Age (Year))	Definition of Age-Related Cataract	Age-Related Cataract Type	Multivitamin/Mineral Supplements Investigated	Confounding Variables Adjusted	Study Quality
Mares-Perlman 2000 (USA) [8]	The Beaver Dam Eye Study	5	Population-based adults (3684, 43–86)	The Wisconsin Age-Related Cataract Grading System	Any type Nuclear Cortical PSC	Block FFQ: multivitamin/mineral supplementation questionnaire (four levels)	Age, sex, smoking, diabetes, hypertension, BMI, UV-B exposure, and hat use in teen years	High
Klein 2008 (USA) [9]	The Beaver Dam Eye Study	15	Population-based adults (4926, 43–86)	The Wisconsin Age-Related Cataract Grading System	Nuclear Cortical	Multivitamin/mineral supplementation questionnaire (yes or no)	Age, sex, smoking, diabetes, hypertension, BMI, UV-B exposure, and hat use in teen years	High
Kuzniarz 2001 (Australia) [10]	Blue Mountains Eye Study	10	Population-based sample (2873, 49–97)	The Wisconsin Age-Related Cataract Grading System	Nuclear Cortical PSC	Multivitamin/mineral supplementation questionnaire (four levels)	Age, sex, hypertension, smoking, diabetes, education, and use of oral and inhaled steroids	High
Rautiainen 2010 (Sweden) [11]	Swedish Mammography Cohort	8	Population-based women (24,593, 49–83)	National registration	Surgery	Multivitamin/mineral supplementation questionnaire (yes or no)	Age, waist circumference, smoking, alcohol consumption, steroid medication use, educational level, and hormone replacement therapy use	Moderate
Zheng 2013 (Sweden) [12]	The Cohort of Swedish Men	8.4	Population-based men (31,120, 45–79)	National registration	Surgery	Multivitamin/mineral supplementation questionnaire (yes or no)	Age, smoking, abdominal obesity, educational level, history of hypertension, corticosteroid use, alcohol intake, and fruit and vegetable intake	Moderate
Milton 2006 (USA) [13]	The Age-Related Eye Disease Study	6.3	Clinic-based adults (4596, ≥45)	Medically diagnosed by slit-lamp and retroillumination photographs graded on a decimal scale	Any type Nuclear Cortical PSC	Multivitamin/mineral (Centrum^®^) supplementation questionnaire (yes or no)	Age, gender, race, smoking, education, lens status, AREDS treatment, and propensity score	Moderate
Leske 1998 (USA) [14]	The Longitudinal Study of Cataract	4.8	Clinic-based population (764, ≥40)	LOCS III cataract classification system	Nuclear	Block FFQ: multivitamin/mineral supplementation questionnaire (three levels)	Age, gender, race, education, current smoking status, coexisting cortical and posterior subcapsular opacities at baseline.	Moderate
Seddon 1994 (USA) [15]	The Physicians’ Health Study	5	US male physicians (17,744, 40–84)	Self-report, confirmed by review of medical records	Any type Surgery	Multivitamin/mineral supplementation questionnaire (yes or no)	Age, randomized treatment assignment, diabetes, hypertension, obesity, alcohol consumption, physical activity, smoking, parental history of myocardial infarction, and high cholesterol.	Moderate
Hankinson 1992 (USA) [16]	The Nurses’ Health Study	8	Female registered nurses (50,828, 45–67)	Self-report, confirmed by medical record review and ophthalmologists	Surgery	Semiquantitative FFQ: multivitamin/mineral supplementation questionnaire (five levels)	Age, time period, diabetes, energy intake, smoking, Quetelet’s index, area of residence, number of physician visits, and aspirin use.	Moderate
Chasan-Taber 1999 (USA) [17]	The Nurses’ Health Study	12	Female registered nurses (47,152, ≥45)	Self-report, confirmed by medical record review and ophthalmologists	Surgery	Multivitamin/mineral supplementation questionnaire (five levels)	Age, time period, diabetes, smoking, BMI, area of residence, number of physician visits, aspirin use, calories, carotene intake, and alcohol consumption.	Moderate
Taylor 2002 (USA) [18]	The Nurses’ Health Study	13–15	Nondiabetic women (492, 53–73)	LOCS III cataract classification system	Cortical PSC	Expanded FFQ: multivitamin/mineral supplementation questionnaire (four levels)	Age, smoke, hypertension, BMI, sun light exposure, and alcohol consumption.	Moderate
Jacques 2001 (USA) [19]	The Nurses’ Health Study	13–15	Nondiabetic women (478, 53–73)	LOCS III cataract classification system	Nuclear	5 FFQ: multivitamin/mineral supplementation questionnaire (four levels)	Age, smoking, hypertension, BMI, sunlight exposure, and alcohol consumption.	Moderate

PSC = posterior subcapsular cataract; LOCS = Lens Opacities Classification System; FFQ = food-frequency questionnaire; BMI = body mass index.

**Table 2:** Randomized controlled trials evaluating multivitamin/mineral supplementation and its association with the low risk of age-related cataract.

Trials	Study Name	Follow-Up Time (year)	Population (Sample Size, Age (year))	Definition of Age-Related Cataract	Definition of Cataract	Intervention	Control	Confounding Variables Adjusted	Study Quality
Maraini 2008 (USA) [46]	Clinical Trial of Nutritional Supplements and Age-Related Cataract Study	9.0	Population-based adults (1020, 55–75)	A modification of the AREDS lens-grading system	Any type Nuclear Cortical PSC Surgery	Multivitamin/mineral supplements	Placebo	Gender, smoking status, and history of steroid use.	High
Sperduto 1993 (China) [47]	The Linxian cataract study	5–6	Population-based adults (2141, 45–74)	LOCS II cataract classification system	Nuclear Cortical PSC	Multivitamin/mineral supplements	Placebo	Not reported.	Moderate

FFQ = food frequency questionnaire; PSC = posterior subcapsular cataract; LOCS = Lens Opacities Classification System; AREDS = the Age-Related Eye Disease Study.

### 3.2. Multivitamin/Mineral Supplements and the Risk of Nuclear Cataracts

Five prospective cohort studies and two RCTs investigated the association between the use of multivitamin/mineral supplements and the risk of nuclear cataract development. All the cohort studies demonstrated that participants receiving between 4.8 and greater than 10 years of multivitamin/mineral supplements had a significant (20%–43%) reduction in the incidence of nuclear cataracts compared with those not receiving any supplementation [8,9,10,13,14,19]. Three studies showed a significant or nearly significant trend towards a decrease in nuclear cataract risk with increased duration of multivitamin/mineral supplementation (*p* = 0.04, *p* = 0.02, and *p* = 0.08, respectively) [8,10,19].

**Figure 1:**
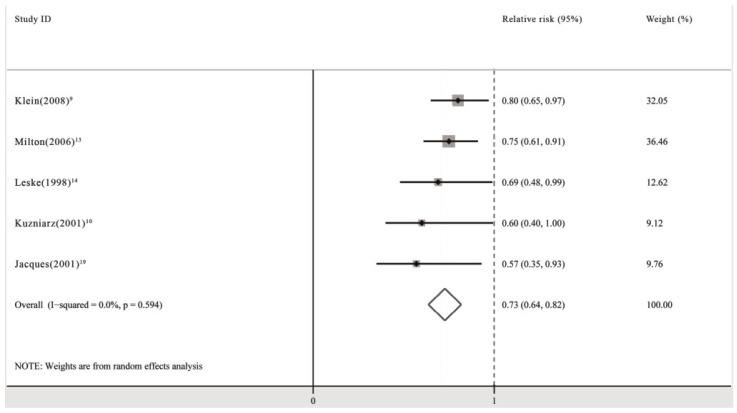
Pooled relative risk (95% CI) of nuclear cataracts comparing the longest duration of multivitamin/mineral supplementation with no supplementation.

**Figure 2:**
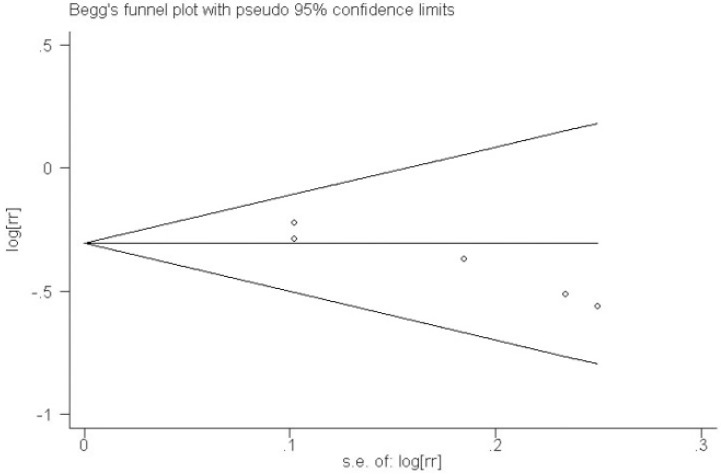
Funnel plot of the relative risk (for the longest duration of multivitamin/mineral supplementation *versus* no supplementation) *versus* the standard error of the log relative risk for studies evaluating nuclear cataracts.

The pooled analysis of these cohort studies revealed that the use of multivitamin/mineral supplements significantly decreased the risk of nuclear cataracts (RR: 0.73; 95% CI: 0.64, 0.82; *P* for heterogeneity: 0.594; *I*^2^: 0.0%) (Figure 1). Begg’s tests revealed no publication bias (*p* = 0.086). The funnel plots showed no asymmetry (Figure 2).

When subgroup analyses including population-based study group (RR: 0.74; 95% CI: 0.56, 0.92; *P* for heterogeneity: 0.249; *I*^2^: 24.8%) [9,10], high quality study group (RR: 0.74; 95% CI: 0.56, 0.92; *P* for heterogeneity: 0.249; *I*^2^: 24.8%) [9,10], and female group (RR: 0.57; 95% CI: 0.28, 0.86) [19] were performed, the pooled RR did not change significantly.

One RCT performed in the USA showed that participants assigned to approximately 9 years of multivitamin/mineral supplements had a significantly lower (34%) incidence of nuclear cataracts than those given a placebo (RR: 0.66; 95% CI: 0.50, 0.88) [46].

Another RCT performed in China demonstrated a significant 43% reduction in the prevalence of nuclear cataracts for persons aged 65 to 74 years who received approximately 5 years of multivitamin/mineral supplements (RR: 0.57; 95% CI: 0.36, 0.90); however, there was no significant reduction for persons aged 45 to 65 years (RR: 1.28; 95% CI: 0.76, 2.14) [47].

### 3.3. Multivitamin/Mineral Supplements and the Risk of Cortical Cataracts

Four prospective cohort studies and two RCTs investigated the association between the use of multivitamin/mineral supplements and the risk of cortical cataract development. Two population-based cohort studies showed that patients who received multivitamin/mineral supplementation for more than 10 years had a significant (23%–30%) reduction in the incidence of cortical cataracts compared with those not receiving supplementation [9,10]. These studies also showed significant trends towards a decrease in cortical cataract risk with increased duration of multivitamin/mineral supplementation (*p* = 0.002; *p* = 0.03, respectively) [8,10]. The other two cohort studies showed no significant beneficial effect of multivitamin/mineral supplements [13,18].

**Figure 3:**
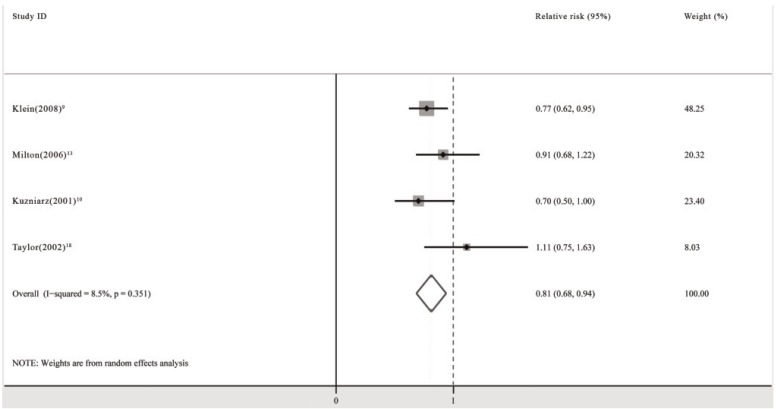
Pooled relative risk (95% CI) of cortical cataracts comparing the longest duration of multivitamin/mineral supplementation with no supplementation.

The pooled analysis revealed that the use of multivitamin/mineral supplements significantly decreased the risk of cortical cataracts (RR: 0.81; 95% CI: 0.68, 0.94; *P* for heterogeneity: 0.351; *I*^2^: 8.5%) (Figure 3). Begg’s tests revealed no publication bias (*p* = 0.734). The funnel plots showed no asymmetry.

When subgroup analyses including population-based study group (RR: 0.75; 95% CI: 0.61, 0.89; *P* for heterogeneity: 0.647; *I*^2^: 0.0%) [9,10] and high quality study group (RR: 0.75; 95% CI: 0.61, 0.89; *P* for heterogeneity: 0.647; *I*^2^: 0.0%) [9,10] were performed, the pooled RR did not change significantly. The female subgroup analysis showed no significant beneficial effect of multivitamin/mineral supplements (RR: 1.11; 95% CI: 0.67, 1.55) [18].

One RCT performed in the USA demonstrated a nearly significant 22% reduction (RR: 0.78; 95% CI: 0.60, 1.02) in the prevalence of cortical cataracts for persons assigned to approximately 9 years of multivitamin/mineral supplementation compared with those assigned to the placebo [46].

Another RCT performed in China demonstrated no significant reduction in the prevalence of cortical cataracts for persons assigned to approximately 5 years of multivitamin/mineral supplements compared with those assigned to the placebo (RR: 1.05; 95% CI: 0.88, 1.26) [47].

### 3.4. Multivitamin/Mineral Supplements and the Risk of Posterior Subcapsular Cataracts

Three prospective cohort studies and two RCTs investigated the association between the use of multivitamin/mineral supplements and the risk of PSC development. All cohort studies demonstrated that multivitamin/mineral supplements did not significantly reduce the incidence of PSC in patients who received between 6.3 and more than 10 years of multivitamin/mineral supplements compared with those receiving no supplementation [8,13,18]. One study showed no significant trend towards a decrease in PSC risk with increased duration of multivitamin/mineral supplementation (*p* = 0.79) [8].

**Figure 4:**
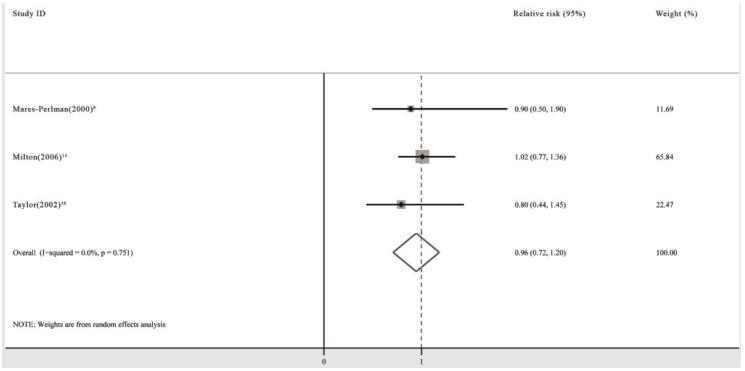
Pooled relative risk (95% CI) of posterior subcapsular cataracts comparing the longest duration of multivitamin/mineral supplementation with no supplementation.

The pooled analysis revealed that the use of multivitamin/mineral supplements produced no significant reduction in the risk of PSC (RR: 0.96; 95% CI: 0.72, 1.20; *P* for heterogeneity: 0.751; *I*^2^: 0.0%) (Figure 4). Begg’s tests revealed no publication bias (*p* = 1.00). The funnel plots showed no asymmetry.

When subgroup analyses including population-based study group (RR: 0.90; 95% CI: 0.20, 1.60) [8], high quality study group (RR: 0.90; 95% CI: 0.20, 1.60) [8], and female group (RR: 0.80; 95% CI: 0.29, 1.31) [18] were performed, the pooled RR did not change significantly.

One RCT performed in the USA demonstrated a significant increase (RR: 2.00; 95% CI: 1.35, 2.498) in the prevalence of PSC for persons assigned to approximately 9 years of multivitamin/mineral supplementation compared with those assigned to the placebo [46].

Another RCT performed in China demonstrated no significant reduction in the prevalence of PSC for persons assigned to approximately 5 years of multivitamin/mineral supplementation compared with those assigned to the placebo [47].

### 3.5. Multivitamin/Mineral Supplements and the Risk of Any Type of Cataract

Three prospective cohort studies and one RCT investigated the association between the use of multivitamin/mineral supplements and the risk of developing any cataract. All cohort studies showed that patients receiving between 5 and more than 10 years of multivitamin/mineral supplements had a significant (16%–60%) reduction in the incidence of any cataract compared with those not receiving supplementation [8,13,15]. Two studies showed significant or nearly significant trends towards a decrease in the risk of any cataract with increased duration of multivitamin/mineral supplements (*p* = 0.002 and *p* = 0.06, respectively) [8,15]. One study showed that multivitamin/mineral supplements produced a slightly larger reduction in the incidence of any cataract for patients ≥65 years (OR: 0.4; 95% CI: 0.2, 0.7) than for patients aged 43–64 years (OR: 0.5; 95% CI: 0.3, 0.9) [8].

**Figure 5:**
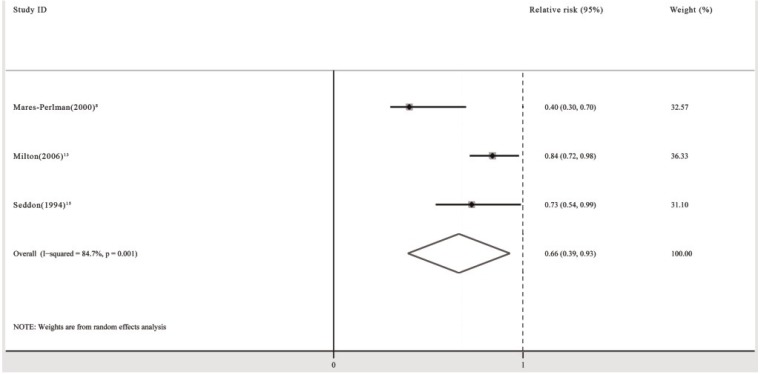
Pooled relative risk (95% CI) of any type of cataract comparing the longest duration of multivitamin/mineral supplementation with no supplementation.

The pooled analysis revealed that the use of multivitamin/mineral supplements significantly decreased the risk of any cataract (RR: 0.66; 95% CI: 0.39, 0.93; *P* for heterogeneity: 0.001; *I*^2^: 84.7%) (Figure 5). Begg’s tests revealed no publication bias (*p* = 0.296). The funnel plots showed no asymmetry.

When subgroup analyses including population-based study group (RR: 0.40; 95% CI: 0.20, 0.60) [8], high quality study group (RR: 0.40; 95% CI: 0.20, 0.60) [8], and male group (OR: 0.730; 95% CI: 0.505, 0.955) [15] were performed, the pooled RR did not change significantly.

One RCT performed in the USA demonstrated that participants assigned to approximately 9 years of multivitamin/mineral supplements had a significantly reduced incidence (by 18%) of any cataract compared with those assigned to the placebo (OR: 0.82; 95% CI: 0.68, 0.98) [46].

### 3.6. Multivitamin/Mineral Supplements and the Risk of Requiring Cataract Surgery

Four prospective cohort studies and one RCT investigated the association between the use of multivitamin/mineral supplements and the risk of requiring cataract surgery. All cohort studies showed that patients receiving multivitamin/mineral supplements had no significant reduction in the incidence of cataract surgery compared with those not receiving supplementation [11,12,15,16,17]. One study showed no significant trend towards a decrease in cataract surgery risk with increased duration of multivitamin/mineral supplements (*p* = 0.30) [16].

**Figure 6:**
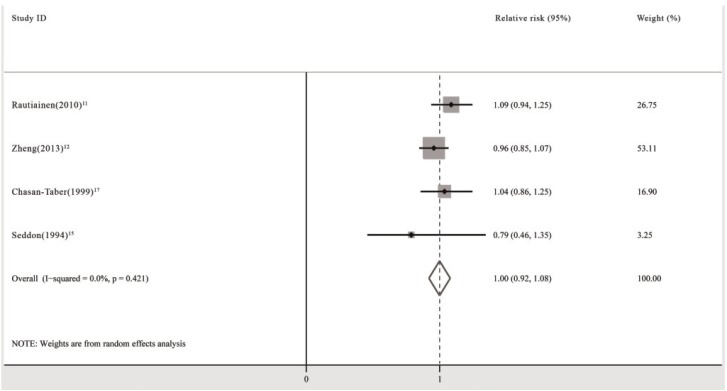
Pooled relative risk (95% CI) of requiring cataract surgery comparing the longest duration of multivitamin/mineral supplementation with no supplementation.

The pooled analysis revealed that the use of multivitamin/mineral supplements had no significant effect on the risk of cataract surgery (RR: 1.00; 95% CI: 0.92, 1.08; *P* for heterogeneity: 0.421; *I*^2^: 0.0%) (Figure 6). Begg’s tests revealed no publication bias (*p* = 1.00). The funnel plots showed no asymmetry.

When subgroup analyses including population-based study group (RR: 1.01; 95% CI: 0.89, 1.14; *P* for heterogeneity: 0.180; *I*^2^: 44.4%) [11,12], female group (RR: 1.07; 95% CI: 0.95, 1.19; *P* for heterogeneity: 0.694; *I*^2^: 0.0%) [11,17], and male group (RR: 0.95; 95% CI: 0.84, 1.06; *P* for heterogeneity: 0.467; *I*^2^: 0.0%) [12,15] were performed, the pooled RR did not change significantly.

One RCT performed in the USA demonstrated no significant reduction (RR: 0.95; 95% CI: 0.71, 1.28) in the risk of cataract surgery for persons assigned to approximately 9 years of multivitamin/mineral supplements compared with those assigned to the placebo [46].

## 4. Discussion

Combining the meta-analysis of the prospective cohort studies and the results of the RCTs, there was a strong inverse association between multivitamin/mineral supplements and age-related cataracts. Additionally, there was a significant trend towards a decrease in cataract risk with increasing duration of multivitamin/mineral supplementation. This beneficial effect was even clearer in older participants.

Nuclear opacities are the most frequent type of cataract and are a well-known biological marker of aging [48]. Multivitamin/mineral supplements have a role in the prevention of nuclear cataracts and may have a role in the prevention of cortical cataracts. The meta-analysis of the selected cohort studies demonstrated that 10 years of multivitamin/mineral supplements had a significantly beneficial effect on cortical cataract prevention. A RCT that included approximately 9 years of follow-up data showed a modest borderline protective association (RR: 0.78; 95% CI: 0.60, 1.02) [46]. The results generated by observational and interventional studies demonstrated that multivitamin/mineral supplements had no or even negative effects on the incidence of PSC. The environmental and genetic factors play greater roles in the risk of PSC development than the oxidative damage caused by aging [49,50]. The results obtained from observational and interventional studies also demonstrated that multivitamin/mineral supplements did not decrease the number of people undergoing surgery for cataract removal. This finding may be explained by not only differences in the levels of severity at which different types of cataract require surgery but also differences in the timing of surgery as decided by the patients and/or surgeons.

The use of dietary supplements is increasingly common in the general population of the United States, Europe, and Asia [39,40,41,42]. In the 1999–2000 National Health and Nutrition Examination Survey, a nationally representative, cross-sectional survey of US health and nutrition, 52% of adults reported taking a dietary supplement within the past month, and 35% reported the regular use of a multivitamin/mineral supplement [39]. Centrum(R) (Wyeth Consumer Healthcare, Madison, NJ, USA) was the most popular multivitamin/mineral supplement, and it contains US Recommended Daily Intake (RDI) levels of many nutrients including 26 vitamins and trace minerals.

Of the included studies, some cohort studies and both RCTs used Centrum(R) as the study supplement [13,46,47], and some cohort studies reported a combination of multivitamins and multiminerals as the study supplement [8,9,11]. Other studies did not definitively indicate whether the multivitamin used in the study included minerals. These cohort studies were population-based, and the use of multivitamin/mineral supplements is common in the general population. Thus, we regarded the included studies as all using multivitamin/mineral supplements as the study supplement, and the current meta-analysis is feasible.

### 4.1. Results in Relation to Other Studies

Over the past two decades, many observational epidemiological studies and RCTs have reported associations between diet and cataract development [8,9,10,11,12,13,14,15,16,17,18,19,20,21,22,23,24,25,26,27,28,29,30,31,32,33,34,35,36,37]. These studies largely examined populations in well-nourished areas, such as the United States, Australia, and other developed Western nations.

Some large-scale cohort studies with a follow-up period of greater than 10 years revealed that a higher dietary intake of vitamin C, vitamin E, lutein/zeaxanthin, carotenoids, riboflavin, folate, niacin, and/or thiamine from food and supplements was associated with a decreased risk of cataract development [16,18,19,20,21,22,23,24,25,26]. Some studies also demonstrated that the long-term use of vitamins C, E, B_12_, or folate supplements alone could significantly decrease the risk of cataract development [10,14,19,27].

RCTs with between 4 and 9.7 years of follow-up data did not demonstrate any beneficial effect of antioxidant vitamins on the risk of cataract development [28,29,30,31,32,33,34,35,36,37]. A recent study based on a meta-analysis of these RCTs also found no evidence that supplementation with antioxidant vitamins (β-carotene, vitamin C, or vitamin E) prevents or slows the progression of age-related cataracts [38]. Other well-designed RCTs, such as AREDS2, also showed that daily supplementation with lutein/zeaxanthin for about 4.7 years had no statistically significant overall effect on rates of cataract surgery or vision loss [51].

There is a discrepancy in the findings between cohort studies and RCTs with regards to the association between antioxidant vitamins and age-related cataracts. The short duration (<10 years) of the interventional trials may explain this discrepancy. Age-related cataracts develop over the course of many years, and antioxidant vitamins may lessen the cumulative minor damage from oxidative stress that occurs over a long period of time. Further long-term RCTs are warranted to verify this hypothesis.

However, some cohort studies and RCTs included in the current study showed that <10 years of multivitamin/mineral supplementation had a beneficial effect on decreasing the risk of age-related cataracts. The meta-analysis of these cohort studies and the results of RCTs were consistent. Potential synergistic effects of vitamin and/or mineral combinations might play a role in the prevention of age-related cataracts.

The Clinical Trial of Nutritional Supplements and Age-Related Cataract Study [46], which was included in our analysis, led to research investigating how long-term multivitamin/mineral supplementation in accordance with the US Recommended Daily Intake (RDI) affected the plasma levels of selected nutrients [52]. Participants assigned to Centrum(R) showed a significant increase in the mean/median plasma levels of vitamin E, β-carotene, vitamin B_12_, folate, and riboflavin compared with participants assigned to the placebo. This result was consistent with the finding of a recent meta-analysis of cohort studies, which revealed that blood levels of certain antioxidants were inversely associated with age-related cataracts [53].

Some RCTs investigated mixtures of oral antioxidant micronutrients, such as β-carotene, vitamin C, and vitamin E, but revealed that these main antioxidant combinations had no apparent effect on the risk of the development or progression of age-related lens opacities [32,33,34,35,36,37].

There is a lack of published studies reporting associations between minerals and cataract development. Although a few case-control studies have suggested that lens opacities were associated with lower levels of iron and selenium [54,55], neither cohort studies nor RCTs provided additional supporting evidence. The beneficial effect of minerals could not be excluded.

In our study, the potential underlying mechanism by which vitamin and mineral supplements decrease the risk of age-related cataracts is still uncertain; however, the widespread public use of commercially available pharmacological doses of multivitamin/mineral supplements for the prevention of age-related cataracts has significance for public health.

### 4.2. Strengths and Weaknesses of the Study

The current meta-analysis has several strengths. First, several high quality cohort studies and RCTs were included. Population-based, large-scale studies allowed us to quantitatively assess the association between multivitamin/mineral supplements and the risk of age-related cataracts. Second, the meta-analysis of observational data was consistent with the results of the RCTs. These two strengths made the conclusions more powerful than those from any individual study. Third, we systematically reviewed and assessed the summarized association between multivitamin/mineral supplements with different types of cataract, including any lens opacity, nuclear opacity, cortical opacity, posterior subcapsular opacity, and cataract surgery. These findings provided a comprehensive view of the association between multivitamin/mineral supplements and age-related cataracts based on the current evidence.

Our study has several limitations. First, only two RCTs were included. Prospective cohort studies provided the most consistent and strongest evidence regarding multivitamin/mineral supplements in the prevention of age-related cataracts. However, the RCTs showed little heterogeneity, and the results of a meta-analysis of RCTs are known to be more precise and convincing than the results of a meta-analysis of observational data [56].

Second, different cataract assessments and supplement durations were used in the included studies, and available data on the individual multivitamin/mineral supplement dosage were rather limited. Nevertheless, we used relative risks for the highest *versus* lowest categories of multivitamin/mineral supplement duration, which could, to some extent, reduce the bias caused by different supplement durations. Furthermore, a time-response analysis in the included studies supports our results.

Third, the designs of observational studies are problematic. Follow-up interviews in cohort studies revealed that recall biases and response errors existed in the detailed information collected from the study population, especially when self-reported diet and supplement questionnaires had been used. Although the analyses of some cohort studies included a number of potential confounding variables, such as smoking, sunlight exposure, and diabetes, the possibility of missing and unknown or unmeasured confounding variables cannot be dismissed.

Finally, with the exception of one RCT conducted in China, all studies were conducted in relatively well-nourished populations in the United States and other developed Western countries. There was a lack of studies performed in poorly nourished populations in developing areas. The results were not comprehensive and are not generalizable to other countries with undernourished populations. Moreover, possible publication bias could occur because we did not include articles not in English. The number of large cohort studies and RCTs in non-English countries or areas is limited.

## 5. Conclusions

Commonly used multivitamin/mineral supplements in this systematic review demonstrated a significantly beneficial effect in decreasing the risk of age-related cataract in well-nourished Western populations. These results seem to be clinically relevant in terms of public health, particularly given the increasing elderly population and increasing life expectancy, and are strictly concordant with current guidelines and recommendations from US Recommended Daily Intake levels of nutrients.
